# Transcriptomic Profiling of Human Placenta in Gestational Diabetes Mellitus at the Single-Cell Level

**DOI:** 10.3389/fendo.2021.679582

**Published:** 2021-05-07

**Authors:** Yuqi Yang, Fang Guo, Yue Peng, Rong Chen, Wenbo Zhou, Huihui Wang, Jun OuYang, Bin Yu, Zhengfeng Xu

**Affiliations:** ^1^ Changzhou Maternity and Child Health Care Hospital Affiliated to Nanjing Medical University, Changzhou, China; ^2^ International Genome Center, Jiangsu University, Zhenjiang, China; ^3^ Womens Hospital of Nanjing Medical University, Nanjing, China

**Keywords:** gestational diabetes mellitus, placenta, single-cell RNA sequencing, cellular signatures, transcriptomes

## Abstract

Gestational diabetes mellitus (GDM) is associated with an increased risk of adverse pregnancy outcomes. Increasing evidence shows that placentation defects may play important roles in GDM. However, our understanding of the human placenta remains limited. In this study, we generated a comprehensive transcriptomic profile of cellular signatures and transcriptomes in the human placenta in GDM using single-cell RNA sequencing (scRNA-seq), constructed a comprehensive cell atlas, and identified cell subtypes and subtype-specific marker genes. In addition, we investigated the placental cellular function and intercellular interactions in GDM. These findings help to elucidate the molecular mechanisms of GDM, and may facilitate the development of new approaches to GDM treatment and prevention.

## Introduction

Gestational diabetes mellitus (GDM) increases the risk of adverse pregnancy outcomes, such as preterm birth, premature rupture of membranes, macrosomia and fetal distress. GDM also affects the long-term health of the expectant mother and her progeny. Insulin resistance (IR) is considered as the central to the development of GDM. However, the precise mechanisms for the occurrence and development of GDM remain to be elucidated. The placenta is an important organ for material exchange between the fetus and mother, which is crucial to maintain a healthy pregnancy. Some studies have shown that GDM-associated defects in IR were tissue-type-dependent, and that glucose tolerance and insulin tolerance were also impaired in GDM placenta (1). Furthermore, increasing evidence suggested that placental defects might play the important roles in GDM (2), relatedmainlyto fatty acid placental transfer (3), specific hormones (placental lactogen, prolactin, oestradiol, etc.), and factors in the placental microenvironment, including cytokines (4), proteins, genes, miRNAs (5), lncRNAs (6), circRNAs (7). However, at present, our understanding of the placenta remains very limited.

Recently, single-cell RNA sequencing (scRNA-seq) has led to broad insights into the nature of human diseases (8,12). In 2020, Han et al. (13) constructed a scheme for the human cell landscape based on single-cell mRNA sequencing. Previous studies have demonstrated that scRNA-seq can be used to comprehensively characterize cellular heterogeneity, identify known or unknown cell types and placental cell-specific gene signatures, reveal cell subpopulations, and analyzed data on transcription factors and putative intercellular communication patterns (14,17). These studies provided one blueprint for further understanding the roles of placental cells, as well as the pathogenesis of pregnancy-related disorders. However, most of the studies have not involved pregnancy-associated diseases. Currently, only preterm labor (18) and preeclampsia (19) have been investigated. The transcriptomic significance of the human placenta in GDM has not yet been studied.

Here, we applied single-cell RNA sequencing to generate a novel comprehensive transcriptomic profile of cellular signatures and transcriptomes in the human placenta of GDM, and built a comprehensive cell atlas for GDM placenta. Our data provide a rich resource to reveal the molecular mechanisms that underlie the pregnancy risk for GDM patients.

## Methods

### Key Resources Table

Please see **Supplementary Table 1**.

### Sample Collection

Twenty GDM patients and 20 normal pregnant patients were recruited from Changzhou Maternity and Child Healthcare Hospital affiliated to Nanjing Medical University. All of the GDM patients were diagnosed according to Guideline No. 393-Diabetes in Pregnancy (20). In addition, we used the following enrolment criteria: singleton, full-term delivery, caesarean section, age less than 35, and not complicated by other diseases. Pregnant women with obesity or undergoing insulin treatment were excluded. The subjects clinical characteristics are shown in **Supplementary Tables 2** and **3**. Among them, four cases (two GDM and two controls) were used for single-cell RNA sequencing, and other cases were used for validation experiments for immunofluorescence, flow cytometry, and other methods.

After informed consent was provided, placental tissue was collected from subjects during caesarean section. The method of placental tissue retention was as follows. First, for scRNA-seq, after delivery we immediately (within 5 mins) removed a small piece of tissue (about 1.5cm^3^) from a region 25cm away from the umbilical cord insertion. Second, for validation testing, the entire placenta was sent to the Department of Pathology within 30min for dissection by a professional pathologist. Before caesarean section, 5 mL of blood was collected with EDTA.

### Single-Cell RNA Sequencing (scRNA-seq)

#### Preparation of Single-Cell Suspensions

Placental samples were minced on ice into <1-mm^3^ pieces, followed by enzymatic digestion using trypsin. Subsequently, the solution was centrifuged at 300 rcf for 30 sec at room temperature and the supernatant was removed. 1 PBS (calcium- and magnesium-free) containing 0.04% weight/volume BSA (400 g/ml) was added to the supernatant, followed by centrifugation at 300 rcf for 5min. The cell pellet was then resuspended in 1ml red blood cell lysis buffer and incubated for 10min at 4C. After red blood cell lysis, samples were resuspended in 1ml PBS containing 0.04% BSA and filtered over SciencewareFlowmi 40-m cell strainers (VWR). Finally, cell concentration and viability were determined by hemocytometers and Trypan Blue staining.

#### Single-Cell RNA-Seq Library Construction and Sequencing

The scRNA-seq libraries were prepared using Chromium Single Cell 3 Reagent v3 Kits. Single-cell suspensions were loaded on a Chromium Single Cell Controller Instrument (10X Genomics) to generate single-cell gel beads in emulsions (GEMs). Briefly, about 16,00020.000 cells were added to each channel, with a targeted cell recovery estimate of 5,0008,000 cells. After generation of GEMs, single-cell RNA-seq libraries were prepared using the Chromium Single Cell 3Library& Cell Bead Kit (10X Genomics) according to the manufacturers protocol. Libraries were sequenced with an IlluminaNovaseq6000 using high-output 75-cycle kits with apreviously reported read length configuration (21).

#### Single-Cell RNA Sequencing Data Analysis

The Cell Ranger software pipeline (version 3.0) provided by 10X Genomics was used to demultiplex cellular barcodes, map reads to the genome and transcriptome using the STAR aligner, and down-sample reads as required to generate normalized aggregate data across samples, producing a matrix of gene counts versus cells. We processed the unique molecular identifier (UMI) count matrix using the R package Seurat (ver. 2.3.4). Low-quality cells (UMI/gene numbers out of the limit of mean value +/- 2 fold of standard and >10% mitochondrial genes) were excluded.

The top variable genes across single cells were identified using the method described in Macosko et al. (22). Briefly, the average expression and dispersion were calculated for each gene, and genes were subsequently placed into 22 bins based on expression. Principal component analysis (PCA) was performed to reduce the dimensionality in the log-transformed gene-barcode matrices of the top variable genes. Cells were clustered using a graph-based clustering approach and visualized in two dimensions using tSNE. We used likelihood ratio tests that simultaneously assessed changes in mean expression and in the percentage of expressed cells to identify significantly DEGs between clusters. We used the R package SingleR, a novel computational method for unbiased cell type recognition of scRNA-seq, with two reference transcriptomic datasets from the Human Primary Cell Atlas (23) to infer the cell of origin of each of the single cells independently, and to identify cell types.

#### Differentially Expressed Genes (DEGs) Analysis and Enrichment Analysis

Differentially expressed genes (DEGs) were identified using the FindMarkers function (test.use = MAST) in Seurat (24). P value < 0.05 and log2foldchange > 0.58 were set as the threshold for significantly differential expression. GO enrichment and KEGG pathway enrichment analysis of DEGs were performed using R software (R Development Core Team, Vienna, Austria) based on the hypergeometric distribution.

#### Pseudotime Analysis

We determined the developmental pseudotime trajectory using the Monocle2 package (25). The raw count was first converted from a Seurat object into CellDataSet object with the importCDS function in Monocle2. We used the differential GeneTest function of the Monocle2 package to identify ordering genes (qval < 0.01) that were likely to be informative for the ordering of cells along the pseudotime trajectory. Dimensional reduction clustering analysis was performed with the reduce Dimension function, followed by trajectory inference with the order Cells function using default parameters. Gene expression was plotted with the plot gene in pseudotime function to track changes over pseudo-time.

#### RNA Velocity Analysis

We performed RNA velocity analysis using the R package velocyto.R (26) v0.6. The RNA velocity was calculated based on spliced and unspliced transcript reads and estimated using a gene-relative model. The resulting velocity estimates were projected onto the t-SNE embedding obtained in Seurat and the pseudotime space produced by Monocle 2.

#### Cell-Cell Communication Analysis

We used CellPhoneDB (27) (v2.0) to identify biologically relevant ligand-receptor interactions from single-cell transcriptomics (scRNAseq) data. We considered a ligand or receptor to be expressed in a particular cell type if 10% of the cells of that type had non-zero read counts for the ligand/receptor encoding gene. Statistical significance was then assessed by randomly shuffling the cluster labels of all cells and repeating the above steps, which generated a null distribution for each LR pair in each pairwise comparison between two cell types. After running 1,000 permutations, P-values were calculated with the normal distribution curve generated from the permuted LR pair interaction scores. To define cell-cell communication networks, we paired ligand-receptor-expressing cell types. The R packages igraph and circlize were used to display the cell-cell communication networks.

### Immunofluorometric Assay

Frozen placental tissue sections were cut into 45-m pieces and fixed with 1% acetone. After inactivation and sealing, 50100l of fluorescently labelled diluted primary antibodies were separately added and incubated at 37C. After PBS rinsing, 50100 l DAPI solution was added to each plate, and the plates were placed in the dark at room temperature. After sealing with anti-extraction seals, the expression of proteins in the cells was observed by confocal laser scanning (Olympus FV3000, Japan).

### Flow Cytometry Analysis

Placental tissue samples were freshly collected and digested with collagenase I (1 g/mL) and DNAseI (1 g/mL) to dissociate cells from the extracellular matrix. Cells were stained with Fixable Viability Dye and labelled with antibodies against CD45, CD16, CD14, CD56, CD11b, CD206 and CD80, followed by incubation for 30min at 4C. Cells were analyzed by flow cytometry using a Beckman CytoFLEX S. The data were analyzed using FlowJo software.

### Statistical Analyses

All analyses were performed in R (ver. 3.4.3, http://www.R-project.org). Students t-test was performed to analyze differences between the GDM group and control group in age, height at delivery, weight at delivery, gestational age at delivery, blood glucose of OGTT, and NK cell and macrophage frequency in placenta. P-values of 0.05 or less were considered statistically significant.

## Results

### Cellular Composition of Human Placental Cells in GDM

**Figure 1:**
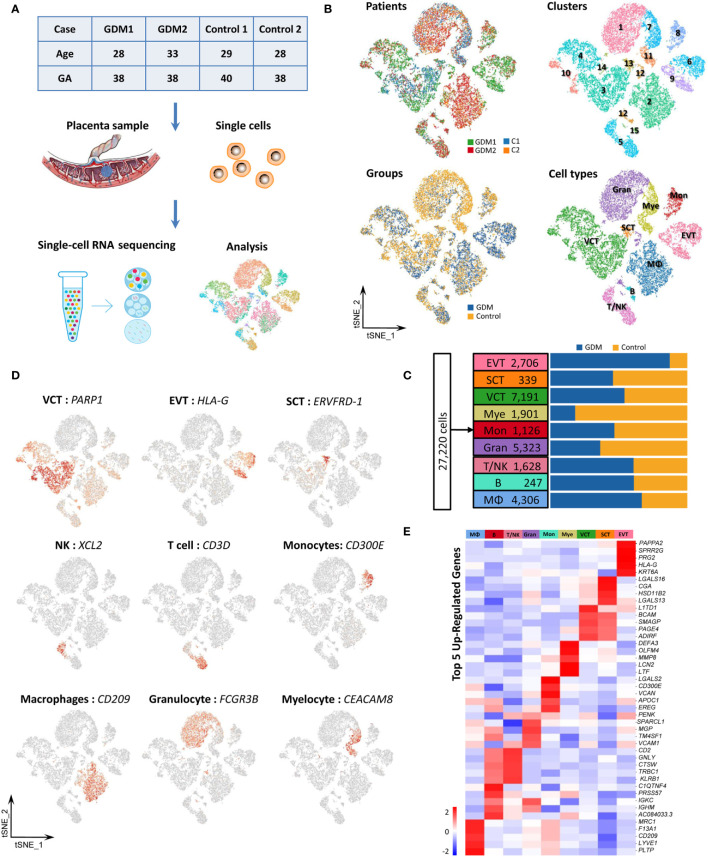
Cellular Atlas for the Human Placenta in GDM. **(A)** The process for single-cell sequencing analysis. **(B)** t-SNE plot of the 27,220 cells profiled, with each cell color-coded to indicate different patients, groups, cell clusters and cell types. **(C)** Comparison of cell numbers for the nine cell types within different groups. **(D)** Expression of marker genes used to identify the nine cell types. **(E)** Heatmap showing the expression signatures for the top five marker genes for each cell type. GDM, Gestational diabetes mellitus; GA, Gestational age at delivery.

To investigate cell-type-specific alterations in GDM at the single-cell level, we performed scRNA-seq on four freshly caesarean-section-delivered placental tissues (two GDM and two normal controls) (**Supplementary Table 2**). After single-cell suspensions were prepared, the entire genome of single cells was sequenced and analyzed using a 10X Genomics microfluidics platform (**Figure 1A**). After quantitative quality control, we obtained a total of 27,220 cells from placental samples (14,591 from GDM samples and 12,629 from control samples). The median number of genes per cell was 1,0541,898, and the median unique molecular identifier (UMI) count per cell was 2,6546,674 (**Supplementary Table 4**). Following previous reports (22), we aggregated transcriptionally similar cells using principal component analysis (PCA). Cells were clustered using a graph-based clustering approach and visualized in two dimensions using t-distributed stochastic neighbor embedding (tSNE). In total, 15 clusters were detected. To identify the different cell types (28) and identified them using cell-type-specific marker genes. Finally, we classified 15 cell clusters into more than nine different cell types (**Figure 1B**). Preliminary analysis revealed no difference in the numbers of clusters and cell types between GDM and normal pregnancy samples. As previously described (19, 29), we found that placental cells differed significantly between groups in terms of composition and abundance (**Figure 1C**). As described further below, we also inferred important differences in cell function.

Placental cell types can be clearly identified using classical cell-type-specific marker genes. Based on these markers, we tentatively identified nine transcriptomically major cell types in the human placenta (**Figure 1C**). **Figure 1D** showed the expression of classical marker genes in each cell type. We also identified the top five marker genes for each cell type, some of which may serve as novel markers (**Figure 1E**). The nine cell types we identified in human placenta by scRNA-seq were villous cytotrophoblast cells [VCT, markers: *PARP1* (15), *MET* (16), *CDH1* (14)], extravillous trophoblast cells [EVT, markers: *HLA-G* (15), *PAPPA2* (16), *MMP11*], syncytiotrophoblastcells [SCT, markers: *ERVFRD-1* (15), *CYP19A1* (30) and *CGA*], T/NK cells [markers: *CD3D* (13), *CD3G*, *GZMA*, *XCL2, CCL5 (* 16 *), GZMK* (31)], B cells [markers: *CD79A* (13), *CD79B*, *CD19*], monocytes [Mon, markers: *CD300E*, *CD244*, *HLA-DRA* (31)], macrophages [M,markers: *CD209*, *CD163* (19), *AIF1* (32)], granulocytes (Gran, markers: *FCGR3B*, *CXCL8*, *MNDA*) and myelocytes (Mye, markers: *TCN1*, *CEACAM8*, *MMP8*). The cell types are summarized in **Table 1** and are discussed in detail below.

**Table 1:** Nine cell types identified in present study.

Cell type	Cluster	Cell numbers	Marker gene
Villous cytotrophoblast cell (VCT)	3,4,10,14	7,191	*CDH1, MET,CCNB2, NRP2, PARP1, INSL4*
Syncytiotrophoblast cell (SCT)	13	339	*CYP19A1, CGA, ERVFRD-1, LGALS13, EGFR*
Extravillous trophoblast cell (EVT)	6,9	2,706	*HLA-G, PAPPA2, MMP2, TGFB1, CXCR6, MMP11*
Granulocyte	1,12	5,323	*FCGR3B, CXCL8, MNDA, SELL*
Myelocyte	7,11	1,901	*TCN1, CEACAM8, S100A8, MMP8, DEFA4, CAMP*,
T/NK cell	5	1,628	*CD3G, CD3D, GZMA, TRBC2, GIMAP2, XCL2, GZMK, IFNG, CCL5, SAMD3*
B cell	12,15	247	*CD79A, CD79B, CD19, FCER2*
Monocytes	8	1,126	*CD14, CD300E, CD244, HLA-DRA, CLEC12A, FCN1*
Macrophages	2	4,306	*CD14, CD68, AIF1, CD163, CD209, CSFIR*

### Single-Cell Transcriptome Profiling of Trophoblast Cells in GDM

**Figure 2:**
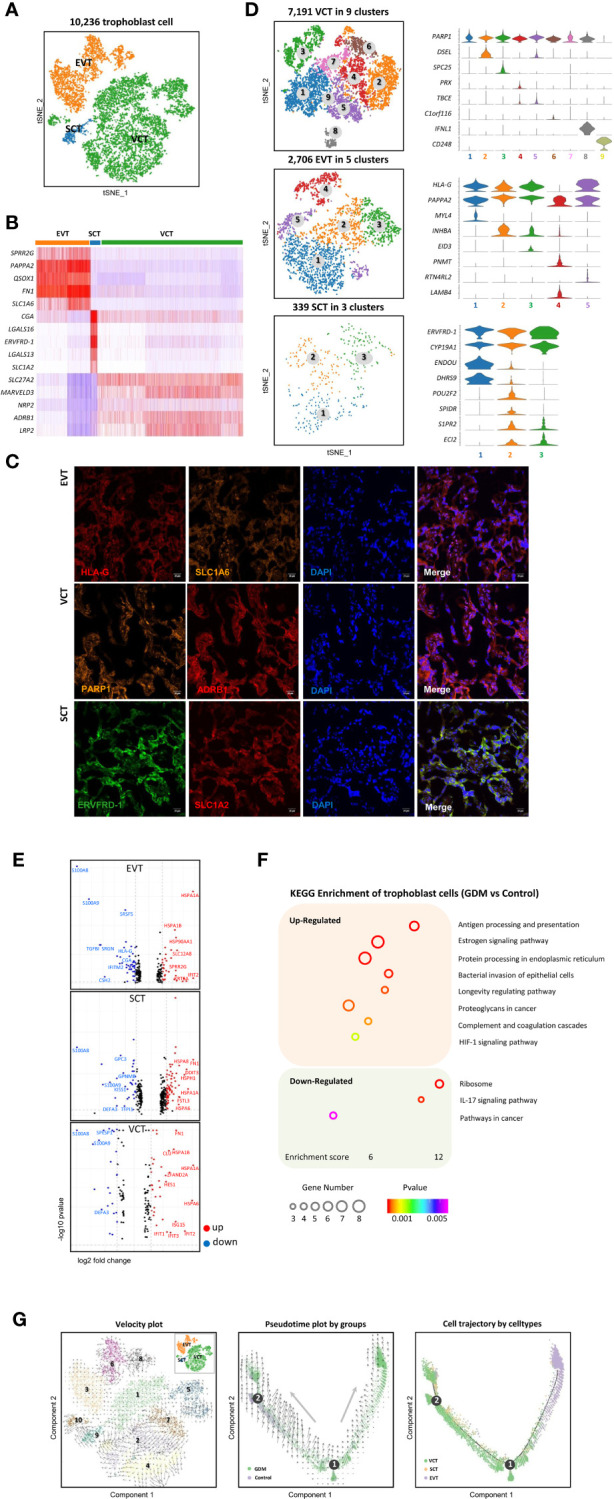
Single-Cell Transcriptome Profiling of Trophoblast Cells. **(A)** t-SNE plot grouping 10,236 trophoblast cells into three subtypes: VCT, EVT and SCT. **(B)** Heatmap showing expression levels for the top five markers for distinguishing VCT, EVT and SCT. **(C)** Three novel markers were identified by immunofluorescence analysis. *SLC1A6*, *ADRB1*, *SLC1A2* can be used to distinguish EVT, VCT and SCT respectively. **(D)** VCT, EVT and SCT were re-clustered into nine, five and three subtypes, respectively. Violin plots show the expression of selected genes within different clusters. **(E)** Volcano plots of differentially expressed genes (DEGs)in VCT, EVT and SCT. Fifty-eight, 75 and 102 DEGs were identified in VCT, EVT and SCT respectively. Red indicates up-regulated genes and blue indicates down-regulated genes. **(F)** Differences in pathway activities scored per cell by KEGG analysis between GDM and control group. **(G)** Pseudotime analysis and RNA velocity of trophoblast cells. The 10,236 trophoblast cells were ordered computationally in terms of RNA velocity (left) and 2D pseudotime trajectory (right).

As previously described, our results showed that trophoblasts were the most abundant cell group in the placenta (**Figure 1C**). Next, we identified gene-expression signatures for trophoblast subtypes with novel markers, explained the specific biological function of GDM trophoblast cells, and reconstructed their differentiation trajectory. We detected 10,236 trophoblast cells, accounting for approximately 37.6% of the total cells. Using classical marker genes (14, 17, 19), all of three types of trophoblast cells were detected, including villous cytotrophoblast cells (VCT) (7,191, 70.3%), extravillous trophoblast cells (EVT) (2,706, 26.4%) and syncytiotrophoblastcells (SCT) (339, 3.3%) (**Figure 2A**). In addition to the classical markers, we identified several potential novel markers for distinguishing among trophoblast subtypes. For example, *SLC1A2* was specifically expressed in SCT, while *SLC1A6* was strongly expressed in EVT (**Figure 2B**). *SLC1A2* and *SLC1A6* are amino acid transporter genes related to the uptake of L-glutamate, L-aspartate and D-aspartate. Placental transfer of amino acids is essential for fetal growth. Moreover, *ADRB1* may be a novel marker for VCT, which has been established as a potential target in GDM (33). The localization of these proteins in cells was confirmed by immunofluorescence analysis (**Figure 2C**).

To discover the alterations in trophoblast subtypes, VCT, EVT and SCT were re-clustered on the t-SNE plot. As shown in **Figure 2D**, re-clustering analysis revealed nine, five and three subtypes, respectively. We next attempted to identify marker genes for each of these clusters. The expression distributions of selected genes in different clusters were shown in violin plots (**Figure 2D**). More detailed classification of trophoblast subtypes may help to reveal their roles in placental development.

Next, 235 differentially expressed genes (DEGs) were identified (pvalue<0.05 and foldchange >2) in trophoblast cells between the GDM and control groups, including 136 up-regulated genes and 99 down-regulated genes (**Figure 2E**). To determine the specific biological functions of GDM trophoblast cells, we performed bioinformatics analysis using gene ontology (GO) analysis and KEGG pathway analysis, as shown in **Figure 2F**. The up-regulated DEGs in GDM were significantly enriched in functions including the estrogen signaling pathway and antigen processing and presentation. The significantly down-regulated DEGs were enriched in the IL-17 signaling pathway, which has important roles in protecting the host against extracellular pathogens.

VCT can differentiate to EVT or SCT during placental development (34, 35). To reconstruct the differentiation pathways, we ordered 10,236 trophoblast cells (VCT, EVT and SCT) computationally with a 2D pseudotime trajectory (36) and RNA velocity (26). Both methods revealed that EVT and SCT originated from VCT (**Figure 2G**), and the differentiation of VCT to SCT seemed to occur earlier. There were also certain differentiation pathways between different cell clusters. For example, clusters 3, 6 and 8 were defined as EVT. RNA velocity predicted a well-ordered differentiation pattern from cluster 3 to cluster 6, and then to cluster 8. Among the clusters of VCT, the cells in clusters 1 and 5 were the primitive cells, and gradually differentiated into clusters 7, 2 and 4. This implies that different trophoblast subtypes may perform different biological functions. However, we did not find a significant difference in the differentiation trajectory of trophoblast cells between GDM and normal-pregnancy samples.

In summary, we identified the gene expression signatures for trophoblast subtypes using novel markers. We also explained the specific biological function of GDM trophoblast cells and reconstructed their differentiation trajectory.

### Immune Cell Subtypes in Human Placenta

As indicated by previous results, immune cell subtypes comprise an important fraction of placental cells. GDM is associated with an impaired maternal immune response. However, few studies have focused on the immune cell composition in GDM (37). We identified various types of immune cells in our specimens, including T/NK cells (1,628), B cells (247), monocytes (1,126) and macrophages (4,306). We also performed re-clustering analysis on immune cells.

**Figure 3:**
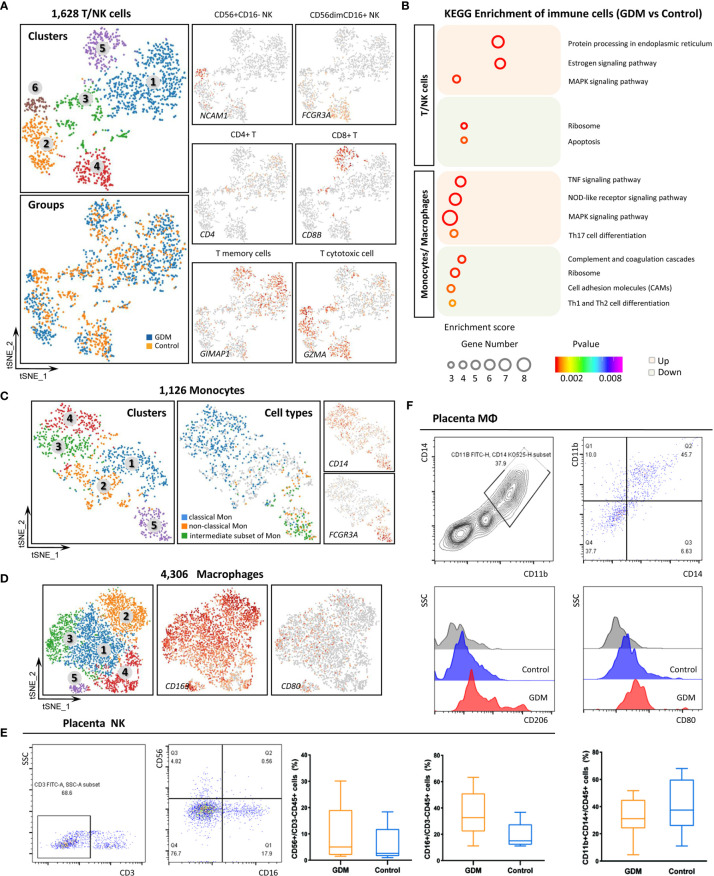
Single-Cell Transcriptome Profiling of Immune Cells. **(A)**In total,1,628 T/NK cells were re-clustered into six clusters. Marker gene expression was used to identify T and NK cell subtypes. **(B)** Representative KEGG analysis of up-regulated and down-regulated DEGs in immune cells from the GDM and control groups. **(C)** Re-clustering analysis of monocytes into five clusters and the expression of marker genes used to identify subtypes. **(D)** t-SNE plot of re-clustered macrophages and expression of marker genes. **(E)** Detection of NK cells in placenta by flow cytometry to confirm the scRNA-seq results in the GDM and control groups. **(F)** Detection of placental macrophages by flow cytometry. The differences in M1/M2 polarization between GDM and normal samples were compared.

We re-clustered the 1,628 T/NK cells into six clusters. Clusters 2, 4, 6 and part of cluster 3 were defined as NK cells with strong expression of *XCL2*, *XCL1*, *CCL5*, *TYROBP* and *KLRD1*. T cells in clusters 1 and 5, and some T cells in cluster 3, were characterized by *CD3D* and *CD3G* expression. However, our results revealed that T cells and NK cells were mixed, so screening filters were used to classify the cells. T cell and NK cell subtypes were identified based on the expression of CD3 (*CD3D*), CD4 (*CD4*), CD8 (*CD8B*), CD56 (*NCAM1*), CD16 (*FCGR3A*), *GIMAP1* and *GZMA* (**Figure 3A**). KEGG analysis of DEGs revealed that the estrogen signaling pathway was significantly up-regulated (**Figure 3B**). Estrogen and its receptor have also been shown to contribute to multiple aspects of T cell function, including reducing T cell activation and proliferation (38).

Monocytes and macrophages at the feto-maternal interface concurrently play important roles throughout pregnancy (39). We grouped the 1,126 monocytes into three subpopulations based on surface marker expression (40, 41): classical monocytes (high *CD14* expression and low CD16 expression), non-classical monocytes (low *CD14* and high CD16) and an intermediate subset of monocytes (high *CD14* and high CD16) (**Figure 3C**). These subtypes have different properties and carry out inflammatory or anti-inflammatory functions. Some studies have indicated that the proportions of monocytes subtypes change throughout pregnancy, but this finding remains controversial (42, 43). Moreover, these previous studies have analyzed maternal peripheral blood rather than placental tissue. We found that 70.2% of monocytes in placental tissue samples belonged to the classical subset, 18.9% to the intermediate subset, and 10.9% to the non-classical subset. The results showed that GDM was associated with an increase in the intermediate subset and non-classical subset and a decrease in the classical subset.

Macrophages (M) can be roughly divided into pro-inflammatory M1-polarized macrophages and anti-inflammatory M2-polarized macrophages, which differ based on the expression of specific surface markers (44). In the present study, 4,306 M were detected, and most of them were M2-polarized. *CD163* and *MRC1* were strongly expressed, while *CD80* expression was low (**Figure 3D**). These results are consistent with previous reports (45). M1 macrophages gradually polarize to M2 throughout pregnancy, and the latter is the predominant type.

To confirm the scRNA-seq results and explore the changes in immune cell populations in GDM placenta, we detected NK cells and macrophages in placenta using flow cytometry (FCM). Twenty fresh placental tissues (10 GDM and 10 controls) were used in the experiment. As shown in **Figure 3E**, the preliminary results indicated that the percentage of NK cells (CD56^+^/CD3^-^CD45^+^) was higher in GDM placenta, and the cytotoxic NK cells (CD16^+^/CD3^-^CD45^+^) also seemed to be present at an increased frequency. We also clearly detected many macrophages in the placenta. The percentage of M (CD11b^+^CD14^+^/CD45^+^) did not significantly differ between GDM samples and normal samples. However, we observed a trend towards enhanced M2 (CD206^+^) polarization and attenuated M1 (CD80^+^) polarization (**Figure 3F**).

### Granulocyte and Myelocyte Subtypes in GDM Placenta

**Figure 4:**
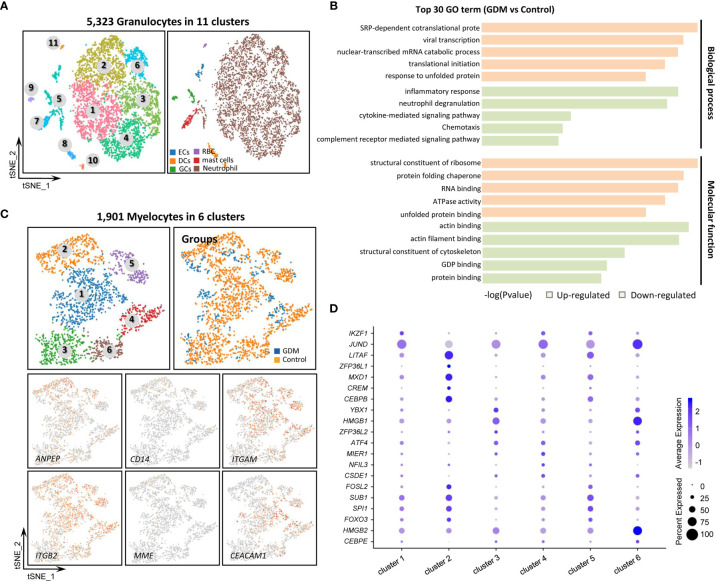
Single-Cell Transcriptome Profiling of Granulocytes and Myelocytes. **(A)** Re-clustering analysis of granulocytes into 11 subtypes. **(B)** Representative GO terms for DEGs between GDM and control group granulocytes. **(C)** Re-clustering analysis of myelocytes into six clusters. Expression of marker genes used to identify subtypes. **(D)** Dotplot showing the top five marker genes for the six myelocyte clusters.

The 5,323 total granulocytes were grouped into 11 separate clusters (**Figure 4A**) based on the expression of specific markers. The cell types detected included neutrophil (*FCGR3B*, *MNDA*), endothelial cells (ECs, *VWF, CDH5*, *PECAM1*), dendritic cells (DCs, *CYBB*), germ cells (GCs, *NOTUM*), T cells (*CD300E*), mast cells (*HDC*) and erythroid-like cells (*HBG1*). However, these cells were more mixed, we only made a simple subtypes distinction in this study. DEGs between the GDM and control groups were identified, and GO and KEGG pathway analyses were performed (**Figure 4B**). GO terms specific to up-regulated DEGs in GDM samples included SRP-dependent co-translational protein, viral transcription and nuclear-transcribed mRNA catabolic process within the biological process category, and structural constituent of ribosome, protein folding chaperone and RNA binding within the molecular function category. Meanwhile, significantly down-regulated genes in GDM samples were enriched mainly in inflammatory response and actin binding.

Myelocytes, a motile cell type with cytoplasmic granules, were also detected in human placenta. The 1,901 total myelocytes were re-clustered into six subtypes (**Figure 4C**). They expressed the CD13 (*ANPEP*), CD18 (*ITGB2*) and *CD55* markers, but did not express the *CD14* and CD10 (*MME*) markers (46). Cluster 3 resembled promyelocytes, with weak CD11b (*ITGAM*) and CD66a (*CEACAM1*) expression. Other clusters were similar to myelocytes. **Figure 4D** shows the top five marker genes within different myelocyte clusters.

### Potential LigandReceptor Interactions in Human Placenta

**Figure 5:**
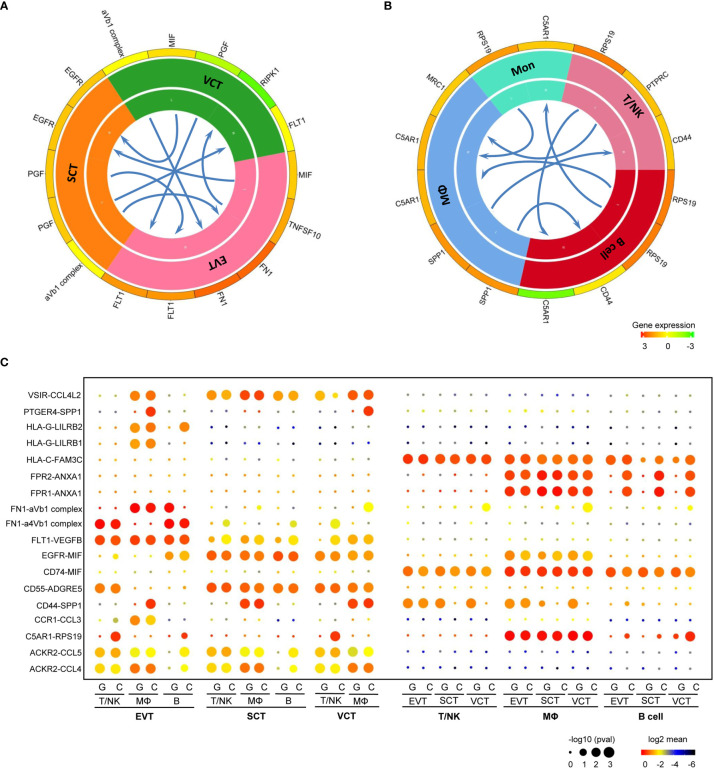
Potential Ligandreceptor Interactions in Human Placenta. **(A)** Intercellular communications among trophoblast cells. **(B)** Intercellular communications among different types of immune cells. **(C)** Comparison of intercellular communications in GDM samples vs. normal samples.

To investigate intercellular communication in human placenta, we visualized average expression levels of the most abundant ligands and their cognate receptors for ligandreceptor pairs in the main cell types. **Figure 5** shows the main interactions between trophoblast subtypes and immune cell types.

Among the three trophoblast subtypes (**Figure 5A**), there were abundant interactions between placental growth factor (PGF) ligand and soluble fms-like tyrosine kinase 1 (FLT1) receptor. PGF was transcribed in SCT and VCT, whereas its receptor, FLT1, was expressed in EVT. MIF-EGFR interaction is another important ligandreceptor relationship; the MIF ligand was restricted mostly to VCT and EVT, and the EGFR receptor to SCT. Within the different types of immune cells (**Figure 5B**), *RPS19*, which encodes ribosomal 40S subunit protein, was expressed in B cells, while its receptor (C5AR1 receptor) was expressed in macrophages. SPP1-CD44 communication was restricted to macrophages, T/NK cells and B cells.

Next, we investigated the intercellular communication between trophoblasts and immune cells in GDM vs. normal samples (**Figure 5C**). There are abundant ligandreceptor interactions between trophoblasts and immune cells in the maternalfetal interface microenvironment, including VEGFB-FLT1, MIF-EGFR, ADGRE5-CD55, CCL3-CCR1 and CCL5-ACKR2. We focused on ligandreceptor pairs whose expression differed significantly between GDM and control samples, and assessed their potential roles in the development of GDM. Surprisingly, the RPS19-C5AR1 ligandreceptor complex, which is restricted to EVT and T/NK cells, was also expressed in the normal placental tissues. However, it was nearly absent in GDM placental samples. RPS19-mediated immunosuppression, initiated by reducing RPS19 or blocking RPS19-C5AR1 interaction, has been shown to inhibit the production of regulatory T cells (47) and may also play a role in inflammation (48) and apoptosis (49). Similarly, SPP1-PTGER1 and SPP1-CD44 pairs exhibited significantly reduced expression in GDM placenta. These pairs play a role in communication between trophoblasts and macrophages. However, the reduced expression in GDM samples was observed mainly in EVT.

Collectively, our results imply that there are complex intercellular communications in the placental microenvironment and characteristic changes in GDM.

## Discussion

Maternal insulin resistance, low-grade inflammation and endothelial cell dysfunction are central features of pregnancies complicated by GDM. Hyperglycemic intrauterine environments affect not only the fetus but also placental development and function. Increasing evidence shows that placental defects may play important roles in GDM. However, our understanding of GDM placental dysfunction remains limited. To our knowledge, this study is the first to describe acomprehensive cell atlas for the GDM placenta with single-cell RNA sequencing. The results provide five noteworthy contributions. First, we describe cell-type-specific alterations in GDM and annotate nine transcriptomically major cell types in the human placenta. Second, we identify several previously unreported marker genes for distinguishing among the three main types of trophoblast and further subtypes. Third, we demonstrate the specific placental function of DEGs in GDM using bioinformatics analysis. Fourth, we explore characteristic changes in immune cells (NK cells and macrophages) in the GDM placenta. Fifth, we report abundant ligandreceptor interactions between trophoblasts and immune cells in the maternal-fetal interface microenvironment. These dysfunctional ligandreceptor interactions may play important roles in the development of GDM. These novel observations provide insight into placental dysfunction in GDM, and will help to reveal the molecular mechanisms of pregnancy risk for women with GDM.

In this study, we focused on the characteristics of trophoblast cells. We not only identified three trophoblast groups (VCT/EVT/SCT) but also detected potential subtypes and corresponding novel markers within each of these groups based on re-clustering analysis. We observed that the subtypes appeared to follow different differentiation pathways. There have been few studies to date on trophoblast subtypes. Liu et al. (14) identified new placental cell subtypes by collecting tissue samples at different stages (the first and second trimesters). They reported three subtypes of VCT and EVT, which do not yet have clear definitions or classifications. Because we do not yet have classical markers for these subtypes, we cannot fully understand their functional characteristics. On the other hand, the differentiation between trophoblast groups and subtypes is a dynamic process that takes place throughout placental development. Therefore, studies carried out in the third trimester may not fully capture the characteristics of trophoblast subtypes. According to KEGG analysis of DEGs, the estrogen signaling pathway and antigen processing and presentation were significantly enriched in GDM specimens. Previous studies reported that GDM could affect the expression of placental ER at the epigenetic level, which plays a key role in energy balance, insulin resistance and trophoblast differentiation (50). Deng etal. also found that the antigen processing and presentation pathway and immune-related genes were closely associated with GDM based on KEGG pathway analysis of differentially methylated genes in omental visceral adipose tissue (51). Conversely, significantly down-regulated genes were involved in the IL-17 signalling pathway, which plays important roles in protecting the host against extracellular pathogens. Although some reports show that GDM patients may have abnormal IL-17 levels, this finding has not yet been verified. The present findings will help to elucidate the function of trophoblasts.

Immune cells are also an important cell group in the placenta. The development of maternal-fetal immune tolerance in the placenta is central to a healthy pregnancy. However, our understanding of the placental immune microenvironment remains incomplete. In this study, we focused on NK cells and macrophages. NK cells are enriched in the early decidua, but gradually decline throughout pregnancy (52). Using scRNA-seq, we confirmed that NK cells remained present in the decidua in late pregnancy. The function of decidual NK cells in late pregnancy may therefore have been overlooked to date, as most studies have focused on peripheral blood NK cells. For example, Chiba etal. reported that NK cells were significantly less abundant in GDM patients, and may have abnormal functions (53). Using FCM, our preliminary findings indicated that there may be an increase in NK cells in the GDM placenta, especially CD16+ cells. Hara et al. (54) also reported that hyperglycemia may promote secretion of inflammatory cytokines and induce production of CD16+ cells, implying that the NK cytotoxicity might increase in the placenta of pregnant women with GDM.

While macrophages play an important role in pregnancy, their frequency and function in GDM placenta remain unknown. Our results show that the proportion of M was not significantly altered in GDM placenta, but that M2 polarization tended to increase. Similarly, Schliefsteiner reported that M2 markers, such as CD206 and CD209, were upregulated in GDM placentae (55). Macrophage-like cells with strong expression of CD163 were significantly more abundant in the chorion and decidua in GDM samples compared with normal controls (56). However, the present findings are preliminary and must be verified by larger-scale follow-up studies. Furthermore, it remains to be determined how to collect placental samples while avoiding heterogeneity.

The human placenta is a dynamic and heterogeneous organ. Our results show that it contains not only a large number of trophoblasts but also many immune cells. These cells do not exist in isolation, and must interact with other cells in their environment to maintain normal placental function. We investigated potential interactions between trophoblasts and immune cells and found that they had many ligandreceptor interactions in the maternalfetal interface microenvironment. The disruption of these interactions may lead to pregnancy complications. For example, preeclampsia is associated with uterine NK cells and extravillous trophoblast cells. NK cell inhibition due to HLA-C2-ligandKIR2DL1-receptor interaction may lead to preeclampsia (57). While the relationships between trophoblasts and immune cells remain incompletely characterized, we discovered several potentially interesting phenomena, including the existence of an RPS19-C5AR1 ligandreceptor complex between EVT and T/NK cells and SPP1CD44 interactions between trophoblasts and macrophages.

We also confirmed that the effects of GDM on the placenta in late gestation were mainly related to placental function rather than structure. This finding is consistent with previous reports (58). We did not find any cell types specific to GDM at the single-cell level in our analysis. The placenta is a highly vascular organ and normal angiogenic function is required for maintenance of pregnancy. In this study, some DEGs related to angiogenesis were found in GDM. However, bioinformatics analysis suggested that they were not the main cause of the GDM placental defects. The results suggested that the most important change in the placental microenvironment in GDM occurs in the immune balance.

It should be noted that this study was preliminary. Although we used clinical samples to verify our results, the small sample size used for scRNA-seq may have been affected by variation due to the placental heterogeneity. Our validation of protein expression and cell function changes between GDM and normal controls was also limited. It remains unclear how changes in placental cells may be caused by GDM or contribute to GDM.

In conclusion, this study was the first to reveal cell-type-specific transcriptomic alterations in GDM placenta at the single-cell level and to explore cell types and cell-type-specific marker genes in the human placenta. In addition, this study demonstrated key features of placental function and cell interactions in GDM. These findings will help reveal the molecular mechanisms of GDM and develop innovative interventions to prevent and treat GDM.

## Data Availability Statement

The data presented in the study (NGS sequencing raw data) were uploaded to GEO repository accession number (GSE173193) (https://www.ncbi.nlm.nih.gov/geo/query/acc.cgi?acc=GSE173193).

## Ethics Statement

The study design and protocol were reviewed and approved by the ethics committee of Changzhou Maternity and Child Health care Hospital affiliated to Nanjing Medical University on 10 January 2020 (NO. 2020160). The patients/participants provided their written informed consent to participate in this study.

## Author Contributions

BY and ZX conceived the study and carried out the assays. YY, FG, and WZ carried out laboratory tests and performed the statistical analysis. YP and HW collected the clinical cases. RC carried out flow cytometry analysis. JOY carried out immunofluorometric assay. YY and BY wrote the manuscript. All authors contributed to the article and approved the submitted version.

## Funding

This study was funded by a project supported by the National Natural Science Foundation of China (81773438), Changzhou Key Laboratory of High-tech Research (CM20193009) and Young Talent Development Plan of Changzhou Health Commission (CZQM2020097).

## Conflict of Interest

The authors declare that the research was conducted in the absence of any commercial or financial relationships that could be construed as a potential conflict of interest. 
