# Supplementary material for: Transcriptomic Profiling of Human Placenta in Gestational Diabetes Mellitus at the Single-Cell Level
Source: Front Endocrinol (Lausanne). 2021 May 7;12:679582. doi: 10.3389/fendo.2021.679582 (PMC8139321; doi:10.3389/fendo.2021.679582)
Supplement: Supplementary file 1 [file Table_1.doc]

**Supplementary table 1. Key Resources of the study**

| **REAGENT or RESOURCE** | **SOURCE** | **PRODUCY NUMBER** |
| --- | --- | --- |
| **Single-cell RNA-sequencing** |  |  |
| Cell Ranger 3.0.0 | 10×Genomics | [https://10xgenomics.com](https://10xgenomics.com/) |
| Seurat 2.3.4 | Nature biotechnology, 2018, 36(5): 411-420. | <https://satijalab.org/seurat/> |
| SingleR 1.3.0 | Nature immunology, 2019, 20(2): 163-172. | <https://github.com/dviraran/SingleR> |
| Monocle 2.9.0 | Nature biotechnology, 2018, 36(5): 411-420. | <https://bioconductor.org/packages/release/bioc/html/monocle.html> |
| SCENIC 1.1.2.2 | Nature methods, 2017, 14(11): 1083-1086. | <https://github.com/aertslab/SCENIC> |
| CellPhoneDB 2.0 | Nature protocols, 2020, 15(4): 1484-1506. | <https://github.com/Teichlab/cellphonedb> |
| Velocyto 0.6 | Nature, 2018, 560(7719): 494-498. | <http://velocyto.org/> |
| GSVA 1.30.0 | BMC bioinformatics, 2013, 14(1): 7. | <https://www.bioconductor.org/packages/release/bioc/html/GSVA.html> |
| **Immunofluorometric assay** |  |  |
| HLA-G | Bioss | bs-0752R-AF647 |
| PARP1 | Bioss | bs-20764R-AF555 |
| ERVFRD-1 | Bioss | bs-15466R-AF488 |
| SLC1A6 | Bioss | bs-13046R-AF555 |
| ADRB1 | Bioss | bs-0498R-AF647 |
| SLC1A2 | Bioss | bs-1751R-AF647 |
| **Flow Cytometry Analysis** |  |  |
| CD45 | Biolegend | 368512 |
| CD56 | Biolegend | 362551 |
| CD14 | Biolegend | 367123 |
| CD86 | Biolegend | 374209 |
| CD80 | Biolegend | 305221 |
| CD11b | Biolegend | 101206 |
| CD16 | Biolegend | 302028 |
| CD3 | Invitrogen | 11-0037-41 |
| Fixable Viability Dye | Invitrogen | 65-0865-18 |
| CD206 | BD | 555954 |
| DNaseⅠ | Sigma | DN25 |
| Collagenase type Ⅱ | Gibco | 17101-015 |

**Supplementary table 2. Clinical characteristics of the subjects who underwent scRNA-seq**

| **Index** | **GDM 1** | **GDM 2** | **Control 1** | **Control 2** |
| --- | --- | --- | --- | --- |
| Age (years) | 28 | 33 | 29 | 28 |
| Height of delivery (cm) | 150 | 163 | 162 | 164 |
| Weight of delivery (kg) | 62 | 83 | 64 | 61.5 |
| Gestational age of delivery (weeks) | 38+1 | 38+2 | 40+4 | 38+3 |
| Nature conceived | Y | Y | Y | Y |
| Singleton pregnancy | Y | Y | Y | Y |
| Primigravid | Y | Y | Y | Y |
| Ethnicity | Han | Han | Han | Han |
| Cesarean section | Y | Y | Y | Y |
| Blood glucose of delivery (mmol/L) | 3.53 | 5.16 | 4.36 | 4.39 |
| OGTT(mmol/L) | 4.46, 8.80, 8.7 | 5.1, 7.9, 9.5 | 3.73, 7.38, 7.02 | 4.54, 7.38, 7.02 |
| Ultrasound abnormality | N | N | N | N |
| Medical diseases | N | N | N | N |
| Family genetic history | N | N | N | N |

Note：

Y: Yes

N: No

OGTT: oral glucose tolerance test,0h, 1h, 2h

**Supplementary table 3. Clinical characteristics of the validated subjects**

| **Index** | **GDM(n=18)** | **Control(n=18)** | ***P*** |
| --- | --- | --- | --- |
| Age (years) | 31.61 ± 4.35 | 31.83 ± 4.62 | 0.883 |
| Height of delivery (cm) | 158.56 ± 4.60 | 157.00 ± 9.78 | 0.546 |
| Weight of delivery (kg) | 69.16 ± 6.62 | 68.29 ± 9.32 | 0.750 |
| Gestational age of delivery (weeks) | 37.65 ± 2.67 | 38.68 ± 1.11 | 0.139 |
| Nature conceived | Y | Y |  |
| Singleton pregnancy | Y | Y |  |
| Primigravid | Y | Y |  |
| Ethnicity | Han | Han |  |
| Cesarean section | Y | Y |  |
| Blood glucose (mmol/L) | 4.79 ± 0.67 | 4.01 ± 0.34 | <0.001 |
| OGTT-1h (mmol/L) | 9.95 ± 1.60 | 6.93 ± 1.13 | <0.001 |
| OGTT-2h (mmol/L) | 8.80 ± 1.58 | 5.95 ± 1.04 | <0.001 |
| Ultrasound abnormality | N | N |  |
| Medical diseases | N | N |  |
| Family genetic history | N | N |  |

Note：

Y: Yes N: No

**Supplementary table 4. Sequencing quality assessment of the study**

| **Index** | **GDM1** | **GDM2** | **Control 1** | **Control 2** |
| --- | --- | --- | --- | --- |
| Estimated Number of Cells | 8430 | 6161 | 7829 | 4800 |
| Mean Reads per Cell | 68529 | 96026 | 67825 | 130068 |
| Median Genes per Cell | 1898 | 1808 | 1524 | 1054 |
| Median UMI counts per Cell | 6537 | 6674 | 5202 | 2654 |
| Sequencing Saturation(%) | 64.6 | 80.7 | 60.7 | 86.4 |
| Q30 Bases in RNA Read(%) | 90.4 | 93.1 | 90.2 | 88.6 |
| Reads Mapped Confidently to Transcriptome(%) | 59.7 | 55.7 | 60.2 | 52.9 |
| Fraction Reads in Cells(%) | 94.6 | 95.0 | 91.0 | 87.7 |

Note: Cell Ranger, a software of 10x genomics, was used to control the quality of sequencing.
